# Manipulation of *Synechocystis* sp. PCC 6803 as a platform for functional identification of genes involved in carotenoid metabolism

**DOI:** 10.1111/pbi.13245

**Published:** 2019-09-11

**Authors:** Tian‐Jun Cao, Lin‐Juan Wang, Xing‐Qi Huang, Yin‐Yin Deng, Li‐En Yang, Shan Lu

**Affiliations:** ^1^ State Key Laboratory of Pharmaceutical Biotechnology School of Life Sciences Nanjing University Nanjing China; ^2^ Jiangsu Marine Fisheries Research Institute Nantong China

**Keywords:** Cyanobacterium, *Synechocystis*, carotenoid, metabolism, enzyme

Carotenoids are widely distributed in nature. They function as light‐harvesting/photoprotective pigments, furnish flowers and fruits with distinct colours, and benefit human health as essential phytonutrients (Rodriguez‐Concepcion *et al*., 2018). All photosynthetic organisms synthesize lycopene and β‐carotene, and higher plants share a more complicated set of carotenoids, including carotenes and their oxygenated derivatives. However, some carotenoids only exist in particular species, such as astaxanthin in the green alga *Haematococcus pluvialis* and lactucaxanthin in lettuce (*Lactuca sativa*) (Rodriguez‐Concepcion *et al*., 2018). The elucidation of enzymes that catalyse their biosynthesis would enable the manipulation of their production by genetic engineering and synthetic biology strategies. It is usually challenging to determine the catalytic activities of these enzymes by *in vitro* assays using recombinant proteins in an aqueous system, because most of their substrates and products are lipid‐soluble. Therefore, *in vivo* assays are also used for functional characterization. The pigment complementation system in *Escherichia coli* is able to synthesize a wide range of carotenoid substrates and has enabled the discovery of a large number of enzymes in this pathway (Cunningham and Gantt, 2007). However, the activities of some enzymes rely on additional cofactors and/or membrane structures. For example, the lycopene β‐cyclase (LCYB) CruA requires a bound chlorophyll *a* molecule, which is absent in *E. coli*, for its activity (Xiong *et al*., 2017). Although functional complementation and/or overexpression in *Arabidopsis* are also widely used for characterizing new enzymes, the complicated and dynamic repertoire of endogenous carotenoids, together with the possible redundancy of enzymes, might mask the functions of the transgenes (Quinlan *et al*., 2007).

Considering that plants exclusively synthesize carotenoids in plastids, which have a cyanobacterial origin, we tested whether the cyanobacterium *Synechocystis* could be engineered as a new platform for functionally characterizing carotenoid metabolic enzymes. Compared with *E. coli*,* Synechocystis* might have an internal environment, such as the availability of cofactors, binding proteins and membrane structures, more similar to that of plastids. On the other hand, *Synechocystis* accumulates only four carotenoids (myxoxanthophyll, zeaxanthin, echinenone and β‐carotene) that all have only β‐ring in their molecules, without any carotenoids that are further modified beyond zeaxanthin or have ε‐rings in their structures. Therefore, *Synechocystis* has a much simpler carotenoid pool than *Arabidopsis* for identifying the functions of exogenous enzymes (Zhang *et al*., 2015). Moreover, the availability of the full genome sequence of *Synechocystis* also provides convenience in genetic manipulation.

We first cloned the kanamycin resistance gene (*Kan* ^*r*^) driven by the *Amp* ^*r*^ promoter (*Amp* ^*r*^ *::Kan* ^*r*^) from pGBK‐T7 as a selection marker. Carotenoid metabolic enzyme genes driven by the promoter of *RbcL* from *Synechocystis* to ensure a high‐level expression were cloned downstream of *Amp* ^*r*^ *::Kan* ^*r*^. When two enzyme genes were to be expressed together, a ribosome‐binding sequence (*RBS*, CTTTAAGAAGGAGATATACC) was used to separate their open reading frames, so that the *RbcL* promoter could drive their expression simultaneously (Thiel *et al*., 2018). Each cassette was further amplified from two ends to incorporate adaptors (GGGGAAAAATCCTCA and GTTTTCCACCAATAC) for facilitating the transformation of *Synechocystis* sp. PCC 6803 by homozygous recombination that replaced the endogenous *CrtO* gene for a β‐carotene ketolase (Xiong *et al*., 2017). Figure 1b shows our designs. *Synechocystis* cells were grown on BG11 plates or in liquid medium at 28 °C under a light intensity of 30 μmol photons/m^2^/s. For selecting transgenic strains, kanamycin at 50 μg/mL was used.

**Figure 1:**
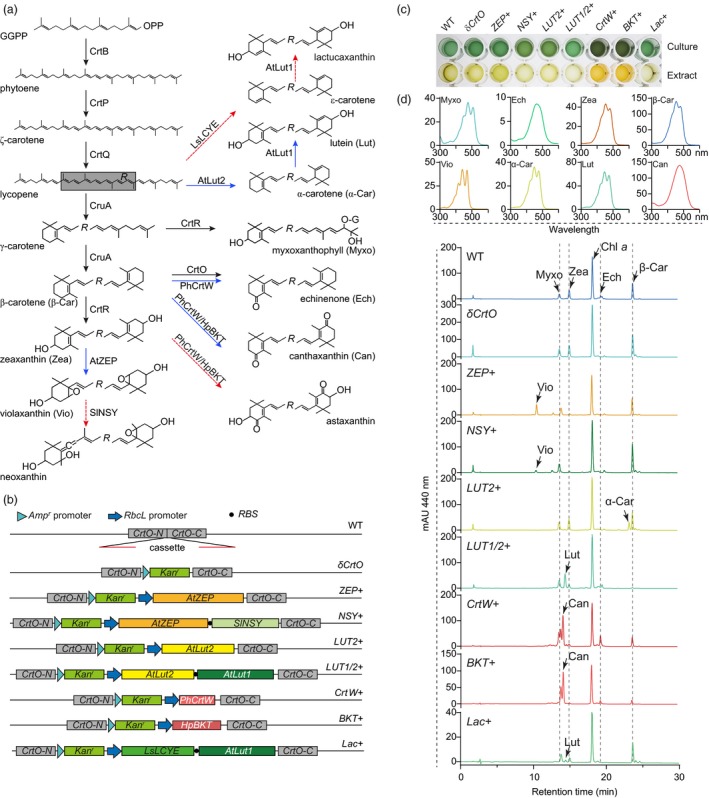
Manipulation of carotenoid metabolism in *Synechocystis*. (a) Scheme of the carotenoid metabolic pathway. Black and blue arrows indicate the reactions catalysed by endogenous and exogenous enzymes, respectively. Red dashes indicate the reactions that exogenous enzymes did not function as in higher plants. Enzymes and their GenBank accession numbers are phytoene synthase (CrtB, P37294.1), phytoene desaturase (CrtP, P29273), ζ‐carotene desaturase (CrtQ, P74306), lycopene β‐cyclase (CruA, BAA18555), β‐carotene ketolase (CrtO, BAA10541) and carotene β‐hydroxylase (CrtR, BAL34979) from *Synechocystis*, zeaxanthin epoxidase (AtZEP, NP_201504.2), lycopene ε‐cyclase (AtLUT2, NP_200513.1) and carotene ε‐hydroxylase (AtLUT1, NP_190881.2) from *Arabidopsis thaliana*, neoxanthin synthase from *Solanum lycopersicum* (SlNSY, Y18297), lycopene ε‐cyclase from *Lactuca sativa* (LsLCYE, AAK07434.1), and β‐carotene ketolases from *Haematococcus pluvialis* (HpBKT, BAA08300.1) and *Paracoccus haeundaensis* (PhCrtW, AAY28417.1). (b) Diagram of the constructs for transforming *Synechocystis* by homologous recombination. Red lines showed the adaptors for replacing a fragment of the endogenous *CrtO* by different cassettes. (c) Representative cultures (at OD_700_ = 2.0) and corresponding methanol extracts of different transgenic strains. (d) HPLC analysis of pigments extracted from different transgenic strains.

*Synechocystis* cultures with different transgenes showed varied colours (Figure 1c). We further analysed their pigments by HPLC (Xiong *et al*., 2017). As expected, when *CrtO* was interrupted by *Amp* ^*r*^ *::Kan* ^*r*^, the transgenic strain was unable to synthesize echinenone (Figure 1d). This further simplified the carotenoid metabolic pathway. Cyanobacteria do not naturally synthesize violaxanthin. However, when the zeaxanthin epoxidase gene from *Arabidopsis* (*AtZEP*) was introduced, the transgenic *ZEP+* strain accumulated violaxanthin instead of zeaxanthin. This demonstrated that *Synechocystis* is suitable to support the function of ZEP.

Because violaxanthin is a substrate for neoxanthin biosynthesis in higher plants (Rodriguez‐Concepcion *et al*., 2018), we further expressed the neoxanthin synthase from *Solanum lycopersicum* (SlNSY) together with AtZEP. No neoxanthin was found in transgenic *NSY+* cells. This is most probably because the function of NSY in higher plants needs two additional proteins, ABA4 and NXD1, which we could not find homologs from the *Synechocystis* genome. However, compared with *ZEP+*, the *NSY+* cells had a lower level of violaxanthin and a higher level of β‐carotene, supporting a primitive function of NSY in catalysing the β‐cyclization of lycopene, as previously reported (Bouvier *et al*., 2000).

Carotenoids with ε‐rings are rarely synthesized in cyanobacteria. To test whether *Synechocystis* can be engineered to produce this group of carotenoids, we introduced the lycopene ε‐cyclase (LCYE) gene from *Arabidopsis* (*AtLut2*) and observed the production of α‐carotene in the corresponding *Lut2+* strain. This confirms that AtLUT2 collaborates with endogenous CruA to catalyse the ε‐ and β‐cyclizations, respectively, of the two open ends of lycopene. We further incorporated the P450‐type carotene ε‐hydroxylase gene from *Arabidopsis* (*AtLut1*), together with *AtLut2*, into the cassette. The transgenic *LUT1/2+* cells accumulated a significant level of lutein, indicating a collaboration of endogenous carotene β‐hydroxylase (CrtR) and exogenous AtLUT1 to hydroxylate both ends of α‐carotene. This indicated a successful construction of the β,ε‐ branch of carotenoid metabolism in *Synechocystis* and demonstrated the feasibility of utilizing this cyanobacterial platform to identify enzymes that catalyse the metabolism of lutein. It is interesting that, compared with the *LUT2* strain, the *LUT1/2+* cells accumulated much lower amounts of β‐carotene and zeaxanthin. This revealed that the expression of additional carotene ε‐hydroxylase resulted in a decreased metabolic flux towards the biosynthesis of carotenoids with two β‐rings in their structures.

We further cloned the β‐carotene ketolase genes from *Haematococcus pluvialis* (*HpBKT*) and the bacterium *Paracoccus haeundaensis* (*PhCrtW*), both of which produce astaxanthin, and expressed each of these genes in *Synechocystis*. Instead of astaxanthin, canthaxanthin and its isomers were accumulated in transgenic cells of both strains. Echinenone was also found in *PhCrtW*‐transformed cell. This showed that both HpBKT and PhCrtW preferentially use β‐carotene as their substrate, and the endogenous CrtR is unable to hydroxylate canthaxanthin to produce astaxanthin, as it was recently demonstrated in rice endosperm (Figure 1a; Rodriguez‐Concepcion *et al*., 2018; Zhu *et al*., 2018). Therefore, the successful production of astaxanthin in *Synechocystis* might need a co‐expression of HpBKT (or PhCrtW) with an additional CrtZ‐type carotene 3,3′‐hydroxylase that uses canthaxanthin as a substrate (Rodriguez‐Concepcion *et al*., 2018).

Carotenoids with two ε‐rings are only found in a few plants, such as lettuce that produces lactucaxanthin. Different from AtLUT2 that catalyses the ε‐cyclization on only one end of lycopene, the LCYE from lettuce (LsLCYE) cyclizes both ends (Cunningham and Gantt, 2007). To test its function in the cyanobacterial system, we co‐expressed *LsLCYE* and *AtLut1* together in our *Lac+* strain. Similar to the *LUT1/2+* strain that expresses *AtLut2* and *AtLut1*, the *Lac+* cells also accumulated only lutein. No carotenoid compounds with two ε‐rings were identified from the products of the *Lac+* strain. This suggests that additional cofactors might be needed for the dual ε‐cyclization function of LsLCYE in cyanobacteria (Cunningham and Gantt, 2001).

Taken together, we demonstrated the feasibility of engineering *Synechocystis* as a new platform for the functional characterization of enzymes involved in carotenoid metabolism and for the screening of unknown factors that interfere with the activities of enzymes such as SlNSY and LsLCYE. Although it is difficult to compare the carotenoid production in *Synechocystis* with that in either *E. coli* or *Arabidopsis*,* Synechocystis* has the advantage of being able to grow both autotrophically and heterotrophically. This means that silencing of its genes for carotenoid biosynthesis is not as crucial as in higher plants for the growth, acclimation, and propagation, and its carotenoid productivity is also released from the restriction of the photosynthetic capability (Fiore *et al*., 2012). Therefore, this *Synechocystis* platform can also be improved as a candidate chassis for the biosynthesis of different carotenoid compounds.

## Author contributions

TJC, LEY and SL designed the experiment. TJC, LJW, XQH and YYD performed the experiments. LEY and SL wrote the manuscript.
